# Regional differences in health care of patients with inflammatory bowel disease in Germany

**DOI:** 10.1186/s13561-015-0067-1

**Published:** 2015-10-16

**Authors:** Ansgar Lange, Anne Prenzler, Oliver Bachmann, Roland Linder, Sarah Neubauer, Jan Zeidler, Michael P. Manns, J.-Matthias von der Schulenburg

**Affiliations:** 1 Leibniz University Hannover, Center for Health Economics Research Hannover (CHERH), Koenigsworther Platz 1, D-30167 Hannover, Germany; 2 Department of Gastroenterology, Hepatology, and Endocrinology, Hannover Medical School, Hannover, Germany; 3 Scientific Institute of TK for Benefit and Efficiency in Health Care (WINEG), Hamburg, Germany

**Keywords:** Inflammatory bowel disease, Quality, Regional differences, Guidelines, Crohn, Ulcerative colitis, I10, I11, I18

## Abstract

**Background:**

The regional availability of specialized physicians is an important aspect in healthcare of patients with IBD. The association between physician density and healthcare is not yet clear. Most studies did not consider district type, which reflects population density. Our research question was, “Do specialist density and district type influence the healthcare of IBD patients in Germany?”

**Methods:**

We combined a claims dataset from a German health insurance fund with population and physician data. Four main aspects were investigated: regular specialist visits, drug therapies, surveillance colonoscopy, and IBD-related hospitalizations. Various regression analyses were performed.

**Results:**

The study cohort was comprised of 21,771 individuals, including 9282 patients with Crohn disease and 12,489 patients with ulcerative colitis. Patients who were living in districts with higher specialist densities were more likely to attend specialist visits on a regular basis. No difference in the frequencies of TNF-alpha inhibitor therapies was found. However, individuals from urban areas were more likely to receive a permanent immunosuppressive therapy with continuous specialist support.

**Conclusions:**

The results revealed that some aspects had positive effects on the probability of implementing healthcare in accordance with pathways and guidelines. No clear evidence of a general healthcare undersupply in rural areas was found.

**Electronic supplementary material:**

The online version of this article (doi:10.1186/s13561-015-0067-1) contains supplementary material, which is available to authorized users.

## Background

Researchers and physicians are constantly searching for possibilities to improve the healthcare situation of patients with inflammatory bowel diseases (IBDs). An important aspect in this discussion is the provision of healthcare resources, especially the regional availability of physicians specialized in the treatment of IBD patients. Principles of optimal healthcare of IBD patients were defined in the course of the development of evidence-based, consented IBD pathways and other guidelines [1–3]. The consortium (representatives of IBD physicians, patient organizations, and insurers) concluded that “IBD patients need a comprehensive, easily accessible, and problem-orientated healthcare” system.

However, whether and how the regional structure, including physician density, influences patient healthcare remain unclear until now. Independent of the indication, these questions have a high public health and political impact worldwide because, for example, the small number of physicians especially in regional areas is often equated with an undersupply of healthcare.

From an empirical point of view, several studies have analyzed the impact of physician density on patient healthcare. Many studies have found a positive association between physician density and healthcare (e.g., less readmissions after heart failure [4], or higher cancer survival [5], or better melanoma prognosis [6]). Others have found no correlation at all [7, 8] or even negative interrelations [9]. To our knowledge, only one study concerning IBD has been conducted previously [10]. According to the results, the overall hospitalization rate for IBDs is similar between residents of high- and those of low-density counties. However, being a resident in counties with high physician density is associated with less complicated disease on hospitalization and lower hospitalization charges for IBD. Nevertheless, this association might also be explained by hospital service quality, which might be higher in urban counties with more specialized hospitals.

The IBD study and most of the other aforementioned studies considered only physician density. The district type, which reflects, among others, the population density and hence the distance within the population, as well as the distance to physicians, may also have an impact on healthcare, especially for chronic conditions such as IBDs. We hypothesized that not only physician density but also district type has an impact on the healthcare of patients with Crohn disease (CD) and ulcerative colitis (UC). Therefore, the overall study question was, “Do specialist density and district type influence the healthcare of IBD patients in Germany?”

With regard to the study question, it is important to define *healthcare* and *good healthcare*. Here, again, the IBD pathways [1] and other guidelines provide orientation. Through our study results, we aimed to scientifically contribute to the discussion of whether patient healthcare is related to physician density and district type and provide special insight into the healthcare situation of IBD patients.

## Methods

### Data source

To address our study question, we used different datasets. We combined a claims dataset from a large German statutory health insurance funding organization (Techniker Krankenkasse [TK], with approximately 7 million insurants in 2008) with population and physician statistical data [11, 12], with the assumption that this approach could provide insights into a potential interrelation between provision quality and regional differences. Claims data are available for the years 2008–2011. The claims database provided anonymized information on patient characteristics and detailed data on inpatient and outpatient care (including diagnoses and operative data), pharmaceuticals, rehabilitation, remedies and aids as well as sick leave payments, but no clinical information. All available information could be merged via an identification number for each individual.

### Patient selection

The selection of the study population was based on diagnostic codes established by the *International Classification of Diseases, 10th Revision, German Modification* (ICD-10-GM). All the patients who had at least either CD (*ICD-10*: K50) or UC diagnosed (*ICD-10*: K51) in the inpatient sector or two confirmed diagnoses in the outpatient sector in 2008 were initially included in the study. This inclusion criteria have been used before in many other studies based on German claims data. Consequently, to prevent the inclusion of patients with potentially incident IBDs, the investigation period chosen was from 2009 to 2011 (hereafter referred to as the “study period”). Due to data protection regulations, more recent and extensive data was not available at the time the analyses were carried out. Patients with an unclassified IBD type (K52, “other noninfective gastroenteritis and colitis”) were excluded.

Patients with no further IBD diagnosis within the study period were also excluded. Since IBD is a chronic disease, this approach should exclude individuals with just a single false diagnosis. Diagnoses of both, CD and UC in a distinct individual let to exclusion to allow the definition of mutually exclusive groups. Furthermore, to be included in the study, patients should be continuously insured in the specific fund plan between 2008 and 2011. Otherwise, no information on e.g. the base-line period (2008) or on other relevant aspects of this study (see below) would have been available. A prerequisite of the analysis is the assignment of the patients to an unambiguous district code. Therefore, patients who changed their place of residence based on official district codes or had no information on district code in their records were also excluded.

### Study design

Aspects from the IBD treatment pathways were used as desired reference points to investigate the regional differences in healthcare of IBD patients. As mentioned in the Background section, we investigated three main aspects from the treatment pathways as follows: 1) regular specialist visits, 2) drug therapies, and 3) surveillance colonoscopy. In addition, we also investigated the presence of IBD-related hospitalizations.

In the following, we will explain these aspects and clarify how they were evaluated using the dataset at hand.

1. Regular specialist visits
  According to the IBD pathways, IBD patients should visit a specialist at least once a year [1]. In this case, gastroenterologists and internists working on an outpatient basis (with or without more than one key focus; hereafter referred to as “specialists”) were said to be specialists (physician identifier nos. 23 and 26) [1]. Therefore, only patients with at least one specialist visit in at least every fifth quarter during the study period were defined as having regular medical checkups.
2. Drug therapies
  Three different main drug therapies were analyzed. The selection was based on consented IBD pathways and other guidelines [1–3] as well as expert opinions. Prescriptions were identified based on the anatomical therapeutic chemical (ATC) classification system.
  - Permanent steroid medication
    The following ATC codes were used to classify steroid medication: A07EA01 and H02AB06, prednisolone; A07EA03 and H02AB07, prednisone; and A07EA06, budesonide.
  - Permanent immunosuppressive therapy
    The following ATC codes were used for immunosuppressive therapy: L04AX01, azathioprine; L01BA01, L04AX03, and M01CX01: methotrexate; and L01BB02, 6-mercaptopurine.
    Steroid dependency or permanent immunosuppressive therapy was considered if patients had a relevant prescription in at least two consecutive quarters during the study period.
  - TNF-α inhibitor therapy
    The following ATC code was used for TNF-α inhibitor therapy: L04AB (etanercept infliximab, afelimomab, adalimumab, certolizumab pegol, golimumab (not approved in 2009)). Owing to the long dosing intervals, patients who had a respective prescription were considered to have a TNF-α inhibitor therapy irrespective of the number of prescriptions.
    Regarding drug therapies, two different aspects were examined. First, we analyzed whether patients received one of the three aforementioned drug therapies. For those patients who received any of the therapies, we additionally analyzed whether the therapy was implemented in accordance with the IBD pathways. The pathways intend that patients who permanently receive one of the three mentioned drug therapies should have continuous specialist support [1]. Therefore, the implementation of the medical therapy is considered to be “according to the IBD pathways” if the patient has at least one specialist visit in the current or following quarter. It should be noted that in principle, a permanent steroid treatment under specialist supervision is deemed as the opposite of good care. However, we could not verify the choice of therapy with our data. Therefore, we determined whether the therapy was implemented under the supervision of a specialist.
3. Surveillance colonoscopy
  Long term UC patients should undergo surveillance colonoscopy on a regular basis according to the IBD pathways [1]. The age at initial manifestation of UC is 15–35 years on average [13]. Following the UC guidelines [14], regular surveillance colonoscopies should be performed at the latest after 15 years of disease duration because of the increased cancer risk. Because the age at diagnosis was not included in the dataset, we assumed that UC patients aged 50 years and older should have had at least two surveillance colonoscopies within the study period (3 years). Information on whether the patients underwent surveillance colonoscopies was based on the German Physicians’ Fee Schedule (EBM) number in the outpatient sector (EBM: 13421 “surcharge colonoscopy”).
4. IBD-related hospitalizations
  We identified all the patients who had at least one hospitalization with a primary diagnosis of CD (K50) or UC (K51) and rated with “had an IBD-related hospitalization.”

We will provide detailed information on the classification of district codes and calculation of physician densities, as they play important roles in the identification of potential regional differences in the aforementioned healthcare aspects. The Federal Institute for Research on Building, Urban Affairs and Spatial Development (BBSR) classified each district (a total of 412 different districts in 2009; the respective district level codes were included in the claims data) according to the four different district types [12]. The differentiation was determined according to the following settlement structures (including population density): 1) autonomous cities, 2) urban areas, 3) rural areas with concentrations, and 4) rural areas without concentrations. Hence, we linked the district level code in the claims data, which represents the place of residence of the respective individual to the respective BBSR district type.

For each of the aforementioned 412 districts, we calculated the specialist density and expressed it as the number of specialists per 10,000 population in each district. Two different densities were calculated using different outpatient physician statistical data as follows: 1) outpatient gastroenterologists + outpatient internist (without more than one key focus) + inpatient gastroenterologists who were authorized for outpatient treatment (counted as 0.5, because half of the time, these physicians were attending inpatients); 2) outpatient gastroenterologists + outpatient internists with permission for screening colonoscopy + inpatient gastroenterologists who were authorized for outpatient treatment (counted as 0.5). The latter definition was only used for the analysis of the surveillance colonoscopy, whereas the former definition was used for all other analyses. The physician data (their profession and district code) were compiled based on information from the National Association of Statutory Health Insurance Physicians (KBV) and the Associations of Statutory Health Insurance Physicians in different federal states.

### Statistical analysis

The aforementioned aspects were analyzed using logistic regression analysis. Therefore, the aspects were dichotomized as described previously. In total, nine different regression analyses were conducted, one for each of the following aspects: “regular specialist visits,” “surveillance colonoscopy,” and “IBD-related hospitalization.” With respect to medication, we conducted two different regression analyses for each aspect. First, we analyzed whether the prescription of the respective medication is dependent on regional differences. Second, for the patients who received such medication, we examined if they received it in accordance with the IBD pathways. The dichotomized variables were each used as the dependent variable in a separate regression model.

Variables for the following items were used as independent variables in the models: sex, age at the onset of the study period (2009), dummy variable accounting for East and West Germany, specialist density, and district type. A dummy variable for CD or UC was used, except for the regression on surveillance colonoscopy. In addition, we used an interaction term to infer how the continuous variable “specialist density” affected the respective dependent variable depending on the magnitude of the categorical variable “district type.” This approach was used to clarify differences in the impact of specialist density on the provision of care across different regions. Coefficients from the logistic regression models were reported as odds ratios. They provide information on how the odds of, for example, a drug therapy according to the IBD pathways increase multiplicatively with a single-unit increase in the independent variable [15]. The odds ratio is similar to relative risk, particularly if a disease is rare. Relative risk is the ratio of the risk for occurrence of a certain event between two groups [16]. Thus, the relative risk provides information on how much risk has increased or decreased from an initial level. However, odds ratio and relative risk are two distinct statistical concepts and are computed in different ways; discrepancies occur only when the initial risk is high [17].

The odds ratio interpretation of logit coefficients cannot be used for interaction terms. Unfortunately, the intuition from linear regression models that the marginal effect of a change in both interacted variables is equal to the marginal effect of the change in just the interaction term does not apply to nonlinear models such as logit models [18]. Both the sign and statistical significance of such effect can be different across observations. Thus, the reported odds ratio and *z*-statistic from the regression output are not substantive for variables that are involved in interactions. Therefore, we calculated and graphed adjusted predictions for these variables. Multicollinearity might be an issue in these models. Therefore, we used the commonly used measures tolerance and variance inflation factor to test for multicollinearity [19]. Furthermore, we conducted Hosmer and Lemeshow’s goodness-of-fit test to assess how good the model fits the data. Given the data source, no ethical approval was required for the study.

## Results

**Fig. 1:**
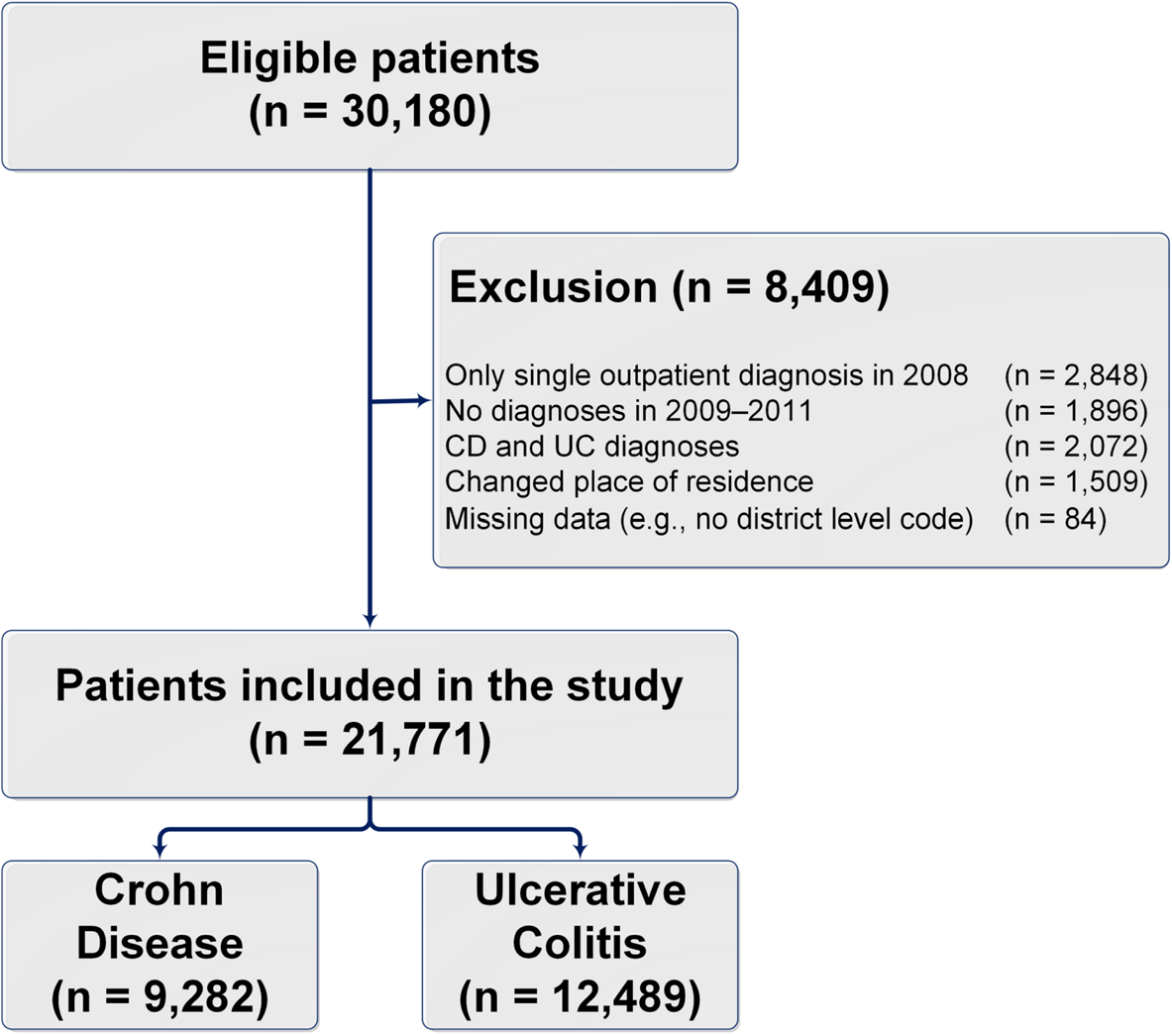
Patient selection flowchart

A total of 30,180 individuals were selected for inclusion based on a relevant diagnosis in the inpatient or outpatient sector. The final study cohort was comprised of 21,771 individuals, including 9282 CD and 12,489 UC patients (Fig. 1). The mean age of the cohort was approximately 50 years and the sex distribution was nearly equal with females comprising 49 % of the cohort. Almost 80 % lived in urban areas or autonomous cities. Further details of the cohort are given in Additional file 1.

In the following, the results of each main aspect are reported separately:

1. Regular specialist visits
  Of all the IBD patients, 21 % (4589) had a regular specialist visit during the study period, including 1855 and 2734 patients from the CD and UC cohorts, respectively (Table 1). The predicted probabilities (Fig. 2) revealed that the patients who were living in areas with higher specialist density were more likely to have specialist visits on a regular basis. This relationship is clearer in both urban district types. However, no relationship was observed in the rural areas with concentrations.

**Table 1:** Descriptive results (main aspects)

		Crohn Disease (CD)	Ulcerative Colitis (UC)	Total
		*n* = 9282	*n* = 12,489	*n* = 21,771
Regular specialist visits		20 %	22 %	21 %
Medication				
Permanent steroid therapy	Received medication	28 %	17 %	22 %
	With continuous specialist support^a^	32 %	37 %	34 %
Permanent immunosuppressive therapy	Received medication	22 %	11 %	16 %
	With continuous specialist support^a^	34 %	38 %	36 %
TNF-α inhibitors therapy	Received medication	7 %	2 %	4 %
	With continuous specialist support^a^	50 %	51 %	50 %
Surveillance colonoscopy (UC patients aged ≥ 50 years (*n* = 6664))		/	9 %	/
IBD-related hospitalization		13 %	5 %	9 %

^a^Only patients who received the relevant medication were considered

**Fig. 2:**
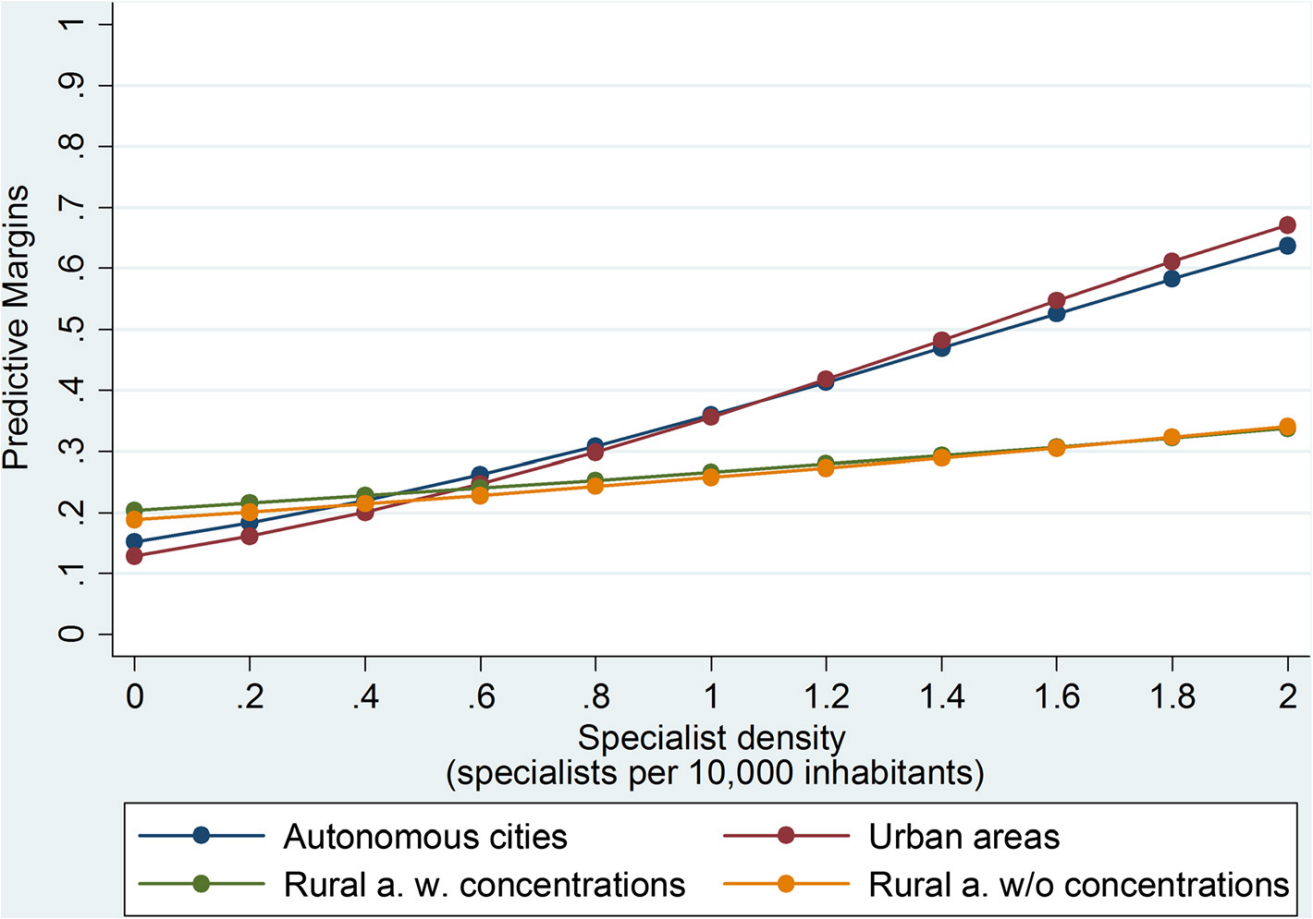
Probability of attending regular specialist visits
2. Drug therapies
  Three different main drug therapies were analyzed. Of the study population, 22 % (4708), 16 % (3434), and 4 % (900) received a permanent steroid medication, permanent immunosuppressive therapy, and TNF-α inhibitor therapy, respectively. The probability of receiving one of the three drug therapies hardly changed, subject to specialist density and district types. Table 1 shows that the proportion of patients who received such drug therapies in combination with regular specialist visits was highest for the TNF-α inhibitor therapy. The plot of the predicted probabilities (Fig. 3) revealed that the probability of receiving a permanent steroid medication or immunosuppressive therapy in combination with regular specialist visits was significantly positively associated with specialist density. Furthermore, this relationship was strongest in the urban district types. However, Fig. 3 shows that the probability of receiving a TNF-α inhibitor therapy in combination with regular specialist visits took a highly different course. As a result, the probability increased and decreased with the higher specialist density in the rural and urban areas, respectively. However, this model is statistically not significant in contrast to all the other regression models in the present study.

**Fig. 3:**
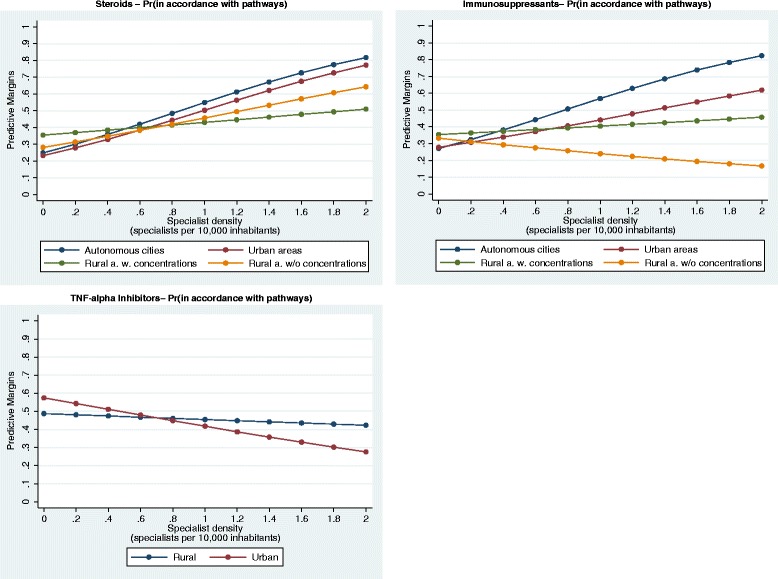
Probability of receiving medication in accordance with the IBD pathways
3. Surveillance colonoscopy
  The group of UC patients, who were 50 years and older, comprised of 6664 individuals. Of the patients, 9 % (587) had regular surveillance colonoscopies during the study period (Table 1). Figure 4 shows that the probability of undergoing regular colonoscopy was mainly independent of the specialist density in the rural areas, whereas in the urban areas, the probability of undergoing regular colonoscopy clearly increased with specialist density. The variation is particularly obvious in autonomous cities, where the probability of undergoing regular colonoscopy increased from 10 % for a specialist density of 0.4 per 10,000 inhabitants to 40 % for a specialist density of 1.4 per 10,000 inhabitants.

**Fig. 4:**
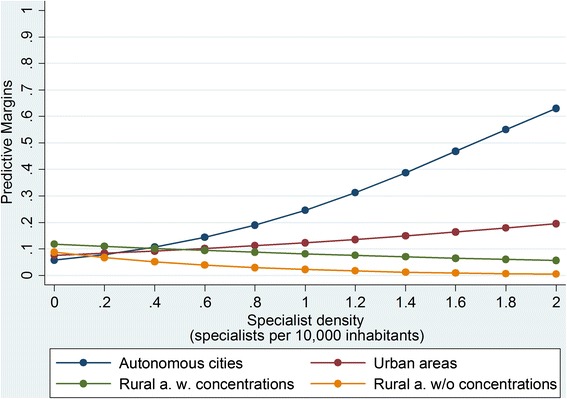
Probability of undergoing regular surveillance colonoscopies
4. IBD-related hospitalizations
  The presence of an IBD-related hospitalization is a surrogate marker of a complicated disease course and is therefore often used as a secondary end point in clinical trials. Approximately 9 % of all the IBD patients had at least one IBD-related hospitalization within the study period (Table 1). Moreover, the probability of having an IBD-related hospitalization was significantly influenced neither by the specialist density nor by the various district types.
  Aside from the main results with regard to specialist density and district types, we analyzed the impact of age, sex, and IBD type (CD or UC) on the aspects of interest using the regression results. These results are provided in Additional file 1.

## Discussion

To our knowledge, this is the first study to analyze the impact of specialist density and district type on the healthcare of IBD patients. The following four main results were derived:

1. The probability of attending *regular specialist visits* increased with specialist density and was more likely for individuals who were living in urban areas than in those who were living in rural areas. One can hypothesize that this result is related to the fact that a higher specialist density may facilitate the access of patients to these specialists. The slight increase illustrated in Fig. 2 clarifies that this might also apply to rural districts. Furthermore, the higher likelihood for urban areas was probably due to the fact that the patient-to-physician distance is shorter than in the rural areas. Therefore, patients visit their physicians more regularly. Our result concurs with that of Busato et al. [20], who analyzed the per capita consultation rates with primary care physicians in Switzerland. They found that the regional density of physicians in independent practice was also significantly associated with annual consultation rates and indicated an associated increase of 0.10 for each additional primary care physician in a population of 10,000 inhabitants.
2. The probability of receiving certain *IBD drugs* was regardless of regional differences. Our results did not indicate general differences in prescription behavior, contrary to the results from the study by Bohlken et al. [8], who examined regional variability in antidementia drug prescriptions in metropolitan and rural regions of Germany. They suggested that the drug coverage was better in rural than in urban areas despite the lower physician density. Furthermore, Windt et al. [21] examined the relationship between prescriptions of TNF-α inhibitors and regional differences based on prescription claims data in 2010. They revealed that the average revenue per insured person was often higher in districts of East Germany. We found no evidence of a higher probability of receiving a TNF-α inhibitor therapy in East Germany. However, we do not focus on the number of prescriptions and the related revenues and the results are not fully comparable.

However, our results revealed that the individuals who were living in urban areas were more likely to receive a permanent immunosuppressive therapy in combination with regular specialist visits. A higher specialist density also had a positive impact on the probability. The pathways define that patients who receive one of the aforementioned drug therapies should have continuous specialist support. Therefore, again, this result could be related to the patients’ easier access to specialists with the increasing specialist density and shorter distances in the urban areas. It is interesting that this insight is not true for TNF-α inhibitor therapies and the probability of receiving such therapies in combination with regular specialist visits diminished with the increasing specialist density in the urban areas. One reason for this discrepancy could be that in the urban areas with higher specialist density, many other internists treated IBD patients, their threshold to prescribe azathioprine or methotrexate presumably being lower that for anti-TNF. Thus, the observed probability of a pathway-consistent anti-TNF therapy decreased.

3) The probability of undergoing *regular surveillance colonoscopies* increased with specialist density. This insight, however, only applies to urban areas. Again, we assume that the higher density facilitated the patients’ access to specialists, particularly in the urban areas. Moreover, in Germany, most surveillance colonoscopies are remunerated from non-budget funds and physicians earn extra money. This fact suggests that our result was likely caused by a supply-induced demand, which is said to be stronger in areas with higher physician density [22–24]. Furthermore, physicians’ time resources might have been scarcer in the rural than in the urban areas [25]. It is noteworthy that only 9 % underwent surveillance colonoscopy on a regular basis. However, this result is in concordance with the result from the study by Kaltz et al. [26]. They revealed that biopsy during colonoscopy that conformed with the guidelines was performed only in 9 % of cases in a colorectal cancer risk group of UC patients in Germany. In addition, other studies have shown that the adherence to surveillance colonoscopy is poor in IBD patients [27–29]. As a sensitivity analysis, the probability of undergoing regular surveillance colonoscopies was analyzed in relation to age. No significant differences were found between the various age classes.
4) The probability of having an *IBD-related hospitalization* was independent of the specialist density and district type. Our result did not indicate general differences in care, in agreement with the results from the study by Ananthakrishnan et al. [10]. They examined whether the availability of physicians is an important determinant of IBD hospitalization in different US counties and found that the overall rate of IBD hospitalizations was similar between residents of high- and those of low-density counties. However, complications were encountered more commonly among hospitalized patients in counties with low physician density.

Other authors have shown that higher physician density may be associated with an increase in healthcare utilization and costs [30]. With regard to the probability of having at least one IBD hospitalization, our results suggest that this does not appear to be the case for IBD. Moreover, factors other than specialist availability may influence the need for hospitalization in IBD patients. In addition, specialist density is merely one marker of availability of regular care. Finally, the effect of specialist density on the probability of having a hospitalization may be small, with other factors playing a more dominant role [10].

Further discussion points related to the results of the impact of age, sex, and IBD-type are provided in Additional file 1.

Our results show that specialist density and district type both have an impact on the healthcare of IBD patients. However, our analyses were mainly based on the German IBD pathways that were formulated as a minimum degree of care for IBD patients. They did not compile data on healthcare oversupply. Therefore, our analyses intend to determine healthcare undersupply only. To summarize, our data do not provide any clear evidence of a general healthcare undersupply (defined by the pathways) for IBD patients in rural areas. However, they reveal that in general, a significantly large proportion of patients are not treated according to the IBD pathways. Particularly, the compliance in terms of regular specialist visits and surveillance colonoscopy is very low. Therefore, patients and providers should be encouraged to improve care. However, against this background, the question of whether the pathways are actually suitable for a comprehensive implementation needs to be answered. If this is not the case, guidelines and pathways should be adapted in a practical and realistic way. Further research is necessary to clarify the impact of differences in quality and quantity of certain treatments on, for example, healthcare cost and quality of life of IBD patients.

Furthermore, we would like to clarify that “adequate” healthcare of IBD patients is not solely related to the frequency of specialist visits. To examine the impact of increased utilization, it would be interesting to link our insights to other utility measures and clinical data, such as quality of life, health status, and well-being of patients. These data are not available in German claims data. Therefore, our study is a first approach to assess the healthcare provided for IBD patients. The German IBD pathways offer more recommendations regarding other aspects that, however, cannot be analyzed by using claims data. Nevertheless, the IBD pathways were developed in interdisciplinary working groups, including patient representatives, and should represent a consensus on the optimal treatment of IBD patients [1].

In the following, we will highlight study limitations with respect to the variables used, the methodology, and the generalizability of the results, even though the data and methodology that we used were the best available to our knowledge. German claims data have some general limitations that are not further discussed here [31]. Concerning the independent variables, we emphasize that we could not obtain any variable that comprises clinical data (e.g. disease activity, severity grade of a disease, symptom scores, laboratory test results, quality-of-life data, smoking status, and body mass index) owing to data protection regulations. Therefore, a classification regarding disease severity was only possible based on drug prescriptions. There might be other differences between our patients that could have biased our results.

The district type variable was also subject to limitations. As stated in the Methods section, the variable reflects, among others, population density and was set by the government. However, it does not necessarily reflect the distances to large cities, and patients living in areas next to large cities might need to travel to visit specialists. If that is the case, our results might have been biased by the small differences between urban and rural areas. Another important variable in our study was specialist density, which accounted for gastroenterologists, internists (without a key focus), and gastroenterological authorized physicians. This definition was subject to uncertainty because it could not precisely foresee which physician was considered as an IBD specialist. Therefore, we conducted our analyses with an alternative definition, taking into account only gastroenterologists and gastroenterological authorized physicians. Our results were changed slightly, but the interpretation remained the same. Thus, we consider our results robust regarding this aspect.

Concerning our dependent variables, we want to highlight that the analysis on regular specialist visits was limited by the nature of the German claims data and reimbursement schemes in the inpatient sector. Services in the inpatient sector are reimbursed based on Diagnosis Related Groups, and data do not comprise the information on whether patients were treated by an IBD specialist or not. Therefore, specialist visits in the inpatient sector were not considered in the present study, and some patients who probably had regular specialist visits might have been coded as not attending regular specialist visits. However, under the assumption that the prevalence of specialist visits in the inpatient sector is similar in urban and rural areas, this problem applies to all patients, and therefore should not bias our results in relative terms.

Furthermore, our definition of a steroid medication might have biased our results. Only prednisolone, prednisone, and budesonide were considered. Although these drugs are the most commonly used in the treatment of IBD, other steroids might have been used as substitutes [32]. In addition, that fact that drug prescriptions are motivated by diseases other than IBD cannot be ruled out. This is especially the case with TNF-α inhibitors, which are also used for other diseases such as rheumatic diseases. This fact might have biased our analysis of drug treatments. Finally, the need for surveillance colonoscopies was closely associated with disease severity. However, information on severity was not available in our data. Patients with extensive disease have an increased risk for developing dysplasia and colorectal cancer and thus should have regular colonoscopies. Patient with only proctitis or proctosigmoiditis do not have an increased risk. Therefore, our results regarding colonoscopies might have been biased owing to the lack of such data.

Our methodological approach also had limitations that need to be considered. We emphasize that the odds ratio interpretation, which is used in the present study, is not equivalent to the relative risk interpretation. Therefore, for example, an odds ratio of 2 does not necessarily correspond to a twofold increase in the risk for a certain exposure. This interpretation would be correct for a relative risk of 2. However, both measures are quite similar under certain conditions, and differences do not lead to a different interpretation in the present study. Furthermore, we included age as a simple linear specification in our models only. This approach may not be appropriate to reveal the effect of age on the outcomes. However, we repeated our analyses to include a squared age term, but our results were unchanged.

Finally, we highlight that the generalizability of our results to all IBD patients in Germany is limited, although the TK is one of the largest sickness funding organization in Germany. This is mainly due to the fact that each sickness fund differs in terms of the group of insured individuals. The comparison of our data with those for the total population of Germany demonstrated that in our data, the individuals who were living in the urban areas and old states of Germany were slightly overrepresented. Therefore, a generalization should be applied with caution. However, no information was available on the geographical distribution of the IBD patients in Germany. Moreover, the estimated number of unreported IBD cases can be assumed to be high in Germany owing to the fact that the disease often progresses over a long period [33]. If these cases are not evenly distributed across the urban and rural areas, our results could be biased. Lastly, it remains unclear the extent to which our results are due to improved health care accessibility or supply-induced demand. This issue merit further research attention to explore the possibilities to improve health care in IBD patients.

## Conclusions

The evidence from this study suggests that factors such as specialist density and district type determine the healthcare provided to IBD patients in terms of certain recommendations from the pathways. However, no clear evidence of a general healthcare undersupply for IBD patients in rural areas was found. Furthermore, age, sex, and IBD type are also important determinants. Finally, the analyses support the application and modification process of the IBD pathways, which should be drawn up in a practical and realistic way.

## Additional file

Additional file 1:
**Additional results and discussion.** (DOCX 31 kb)
