# Supplementary material for: Regional differences in health care of patients with inflammatory bowel disease in Germany
Source: Health Econ Rev. 2015 Oct 16;5:29. doi: 10.1186/s13561-015-0067-1 (PMC4608952; doi:10.1186/s13561-015-0067-1)
Supplement: Additional file 1: — Additional results and discussion. (DOCX 31 kb) [file 13561_2015_67_MOESM1_ESM.docx]

**Electronic Supplementary Material**

Patient characteristics

The study population is described in Table A1.

Table A1: Patient characteristics

|  | Crohn disease (CD) | Ulcerative colitis (UC) | Total |
| --- | --- | --- | --- |
|  | n = 9,282 | n = 12,489 | n = 21,771 |
| Sex |  |  |  |
| Female | 54% | 45% | 49% |
| Male | 46% | 55% | 51% |
| Age (mean ± SD), years | 47.1 **±** 14.5 | 51.9 **±** 14.7 | 49.8 **±** 14.8 |
| Region |  |  |  |
| Western states | 88% | 82% | 84% |
| Eastern states | 12% | 18% | 16% |
| District Type |  |  |  |
| autonomous cities | 31% | 35% | 33% |
| urban areas | 48% | 43% | 45% |
| rural areas with concentrations | 12% | 12% | 12% |
| rural areas without concentrations | 9% | 10% | 10% |
| Specialist density 1  (per 10,000 inhabitants)  (mean ± SD) | 0.38 **±** 0.18 | 0.38 **±** 0.18 | 0.38 **±** 0.18 |
| Specialist density 2  (per 10,000 inhabitants)  (mean ± SD) | 0.26 **±** 0.13 | 0.26 **±** 0.13 | 0.26 **±** 0.13 |

**Further results**

Aside from the main results with regard to specialist density and district types, we analyzed the impact of age, sex, and IBD type (CD or UC) on the aspects of interest using the regression results.

*Sex*

Ceteris paribus, the women were less likely to have regular specialist visits (1), to receive a permanent steroid medication (2a), to receive a permanent immunosuppressive therapy (2b), to receive a TNF-α inhibitor therapy (2c), to have regular surveillance colonoscopies (3), and to have an IBD-related hospitalization (4) than men (Table A2).

*Age*

Ceteris paribus, the older patients were less likely to have regular specialist visits (1), to receive a permanent steroid medication (2a), to receive a permanent immunosuppressive therapy (2b), to receive a TNF-α inhibitor therapy (2c), to have regular surveillance colonoscopies (3), and to have an IBD-related hospitalization (4) than younger patients. Furthermore, if they received a permanent steroid medication (2a_pw) or immunosuppressive therapy (2b_pw), the older patients were less likely to receive these in combination with regular specialist visits (Table A2).

*IBD type*

Ceteris paribus, the UC patients were more likely to have regular specialist visits (1) but less likely to receive a permanent steroid medication (2a), to receive a permanent immunosuppressive therapy (2b), to receive a TNF-α inhibitor therapy (2c), and to have an IBD-related hospitalization (4) than the CD patients. Furthermore, if they received one of the three drug treatments (2a-c_pw), the UC patients were more likely to receive these in combination with regular specialist visits (Table A2).

Table A2: Regression results (selected variables)

|  |  | IBD drugs | | | | | |  |  |
| --- | --- | --- | --- | --- | --- | --- | --- | --- | --- |
|  | Regular specialist visits | Permanent steroid medication | | Permanent immunosuppressive therapy | | TNF-α inhibitors therapy | | Surveillance colonoscopy | Hospitalizations |
|  | 1 | 2a | 2a_pw | 2b | 2b_pw | 2c | 2c_pw | 3 | 4 |
| Variables | OR  (SE) | OR  (SE) | OR  (SE) | OR  (SE) | OR  (SE) | OR  (SE) | OR  (SE) | OR  (SE) | OR  (SE) |
| **UC**  (reference: CD) | 1.141***  (0.0398) | 0.565*** (0.0192) | 1.394***  (0.0896) | 0.531***  (0.0208) | 1.179**  (0.0880) | 0.293***  (0.0231) | 1.073  (0.170) |  | 0.419***  (0.0216) |
| **Female**  (reference: male) | 0.880***  (0.0297) | 0.801*** (0.0270) | 1.153**  (0.0728) | 0.782***  (0.0303) | 1.089  (0.0791) | 0.800***  (0.0560) | 1.000  (0.135) | 0.624***  (0.0578) | 0.837***  (0.0415) |
| East Germany (reference: West Germany) | 1.634***  (0.0754) | 0.726***  (0.0387) | 1.619***  (0.160) | 0.970  (0.0570) | 1.562***  (0.169) | 1.068  (0.110) | 1.126  (0.215) | 0.741**  (0.0965) | 0.897  (0.0686) |
| **Age** | 0.987***  (0.00114) | 0.985***  (0.00114) | 0.980***  (0.00205) | 0.962***  (0.00132) | 0.995**  (0.00245) | 0.953***  (0.00237) | 0.993  (0.00522) | 0.980***  (0.00491) | 0.974***  (0.00167) |
| Observations | 21,771 | 21,771 | 3,186 | 21,771 | 3,283 | 21,771 | 900 | 6,664 | 21,771 |
| Other variables: district type and specialist density; OR: odds ratio; SE: standard error; IBD: inflammatory bowel disease | | | | | | | | | |

***p < 0.01, **p < 0.05, *p < 0.1

**Further Discussion**

In the following, we will provide a discussion on the impact of age, sex, and IBD-type on our results.

Sex and age consistently had a significant impact on the provision of care. In our study, the women were less likely to receive regular surveillance colonoscopies and visit specialists than the men. Moreover, they more rarely received a prescription of one of the relevant drug therapies than did men. Regarding immunosuppressants, a study by Blumenstein et al. [1] obtained contradictory results. It was based on an Internet-based survey conducted between 2005 and 2007, and evaluated sex-related differences in therapeutic management of IBD patients treated in the Rhein-Main region of Germany. The study revealed that the women, who were treated in outpatient services of hospitals, more often received immunosuppressants than the men. No significant differences in gastroenterological practices were found. However, the results showed that TNF-α inhibitor therapy was applied more frequently in men than in woman in hospital outpatient services. This finding is in line with our results. Nevertheless, the patients included in that study represented a small fraction of all the patients with IBD in Germany and were all recruited in one federal state. Furthermore, survey data are distinctly different from claims data and might be biased in other ways. By contrast, our study aimed to analyze the data of patients who were receiving a permanent immunosuppressive therapy. Due to differences in definitions, the results of the previous studies and our study are not perfectly comparable.

In addition, our results correspond to the results obtained by Prenzler et al. [2]. They analyzed healthcare costs and the predictive factors of IBD in Germany, from the perspective of the German statutory health insurance. Ceteris paribus, men incurred significantly higher costs than women. TNF-α inhibitors were identified as the main cost driver. Our results support the previous findings in that they demonstrate that women are less likely to receive one of the relevant drug therapies (including TNF-α inhibitors). One possible reason for this difference between women and men could be the fact that a large proportion of the women in our dataset were of reproductive age. Consequently, physicians might prefer a restrained use of immunosuppressants or *biologicals* if women desired to have a child [1]. However, our analysis did not focus on resource use, and its results should be interpreted with caution concerning this matter.

Furthermore, our study shows that with increasing *age*, the probability diminishes for a healthcare provision in accordance with the IBD pathways and that older patients are less likely to receive a relevant drug therapy and to have an IBD-related hospitalization than younger patients. One can hypothesize that this is related to a decreasing disease severity with increasing age in IBD patients [3, 4]. Furthermore, in older age groups, attention for IBD management might be overshadowed by the other needs of the individuals that come with age. Finally, older patients might have already established a closer relationship with their general physician and neglect specialist visits.

Our results revealed that the *UC* patients were consistently more likely to receive a healthcare supply consistent with the IBD pathways and were less likely to receive certain drugs and to be hospitalized (in relation to IBD) than the *CD* patients. These findings concur with the established knowledge in the field of IBD. Studies have shown that more CD patients require surgical treatment over the course of the IBD than UC patients because of a higher complication rate [5, 6]. The higher incidence of complications might also explain our finding that the UC patients were less likely to receive one of the relevant drug therapies than the CD patients. Furthermore, mesalamine (5-aminosalicylic acid) has become the standard therapy in patients with UC [7]. Thus, UC patients might be less likely to receive one of the drugs that were considered in the present study than CD patients. Moreover, studies show that CD is associated with smoking [8–10]. Meanwhile, smoking is more widespread among the lower than the higher social classes [11]. If it is assumed that higher social classes are assumed to have better conscious awareness of smoking-related illnesses and the treatment methods; this could be an explanation why the UC patients had a higher probability of receiving a healthcare supply consistent with the IBD pathways than the CD patients. However, the social class of the individuals in our data could not be determined; thus, our explanation is tentative.

**References**

1. Blumenstein I, Bock H, Zosel C, Dignass AU, Hartmann F, Zeuzem S, Stein JM, Schroeder O: Are there gender-related differences in the therapeutic management of patients suffering from inflammatory bowel disease? Subgroup analysis of a prospective multicentre online-based. Z Gastroenterol. 2009;doi:10.1055/s-0028-1109647.

2. Prenzler A, Bokemeyer B, Schulenburg JM, Mittendorf T: Health care costs and their predictors of inflammatory bowel diseases in Germany. Eur J Health Econ. 2011;doi:10.1007/s10198-010-0281-z.

3. Cosnes J, Sokol H, Seksik P: How to Identify High-Risk Patients in Inflammatory Bowel Disease? In: Baumgart DC, editor. Crohn's Disease and Ulcerative Colitis. New York, Dordrecht: Springer; 2012. p. 713-725.

4. Charpentier C, Salleron J, Savoye G, Fumery M, Merle V, Laberenne JE, Vasseur F, Dupas JL, Cortot A, Dauchet L, Peyrin-Biroulet L, Lerebours E, Colombel JF, Gower-Rousseau C: Natural history of elderly-onset inflammatory bowel disease: a population-based cohort study. Gut. 2014;doi:10.1136/gutjnl-2012-303864.

5. Riemann J, Fischbach W, Mössner J, Galle P: Gastroenterologie in Klinik und Praxis, 1st ed. Stuttgart, New York: THIEME; 2007.

6. Hurst RD: Surgical Management of Inflammatory Bowel Disease. In: Cohen RD, editor. Inflammatory bowel disease. Diagnosis and therapeutics. Clinical Gastroenterology. Totowa, N.J: Humana Press; 2003. p. 157-199.

7. Baumgart DC: The diagnosis and treatment of Crohn's disease and ulcerative colitis. Dtsch Arztebl Int. 2009;doi: 10.3238/arztebl.2009.0123.

8. Cosnes J, Beaugerie L, Carbonnel F, Gendre JP: Smoking cessation and the course of Crohn's disease: an intervention study. Gastroenterology. 2001;doi:10.1053/gast.2001.23231.

9. Gearry RB, Richardson AK, Frampton CM, Dodgshun AJ, Barclay ML: Population-based cases control study of inflammatory bowel disease risk factors. J Gastroenterol Hepatol. 2010;doi: 10.1111/j.1440-1746.2009.06140.x.

10. Somerville KW, Logan RF, Edmond M, Langman MJ: Smoking and Crohn's disease. BMJ. 1984;doi:10.1136/bmj.289.6450.954.

11. Flint AJ, Novotny TE: Poverty Status and Cigarette Smoking Prevalence and Cessation in the United States, 1983-1993: The Independent Risk of Being Poor. Tobacco Control. 1997;doi:10.2307/20207251.
